# Glass is a Viable Substrate for Precision Force Microscopy of Membrane Proteins

**DOI:** 10.1038/srep12550

**Published:** 2015-07-31

**Authors:** Nagaraju Chada, Krishna P. Sigdel, Raghavendar Reddy Sanganna Gari, Tina Rezaie Matin, Linda L. Randall, Gavin M. King

**Affiliations:** 1 Department of Physics and Astronomy, University of Missouri-Columbia, Columbia, Missouri 65211 USA; 2 Department of Biochemistry, University of Missouri-Columbia, Columbia, Missouri 65211 USA

## Abstract

Though ubiquitous in optical microscopy, glass has long been overlooked as a specimen supporting surface for high resolution atomic force microscopy (AFM) investigations due to its roughness. Using bacteriorhodopsin from *Halobacterium salinarum* and the translocon SecYEG from *Escherichia coli*, we demonstrate that faithful images of 2D crystalline and non-crystalline membrane proteins in lipid bilayers can be obtained on microscope cover glass following a straight-forward cleaning procedure. Direct comparison between AFM data obtained on glass and on mica substrates show no major differences in image fidelity. Repeated association of the ATPase SecA with the cytoplasmic protrusion of SecYEG demonstrates that the translocon remains competent for binding after tens of minutes of continuous AFM imaging. This opens the door for precision long-timescale investigations of the active translocase in near-native conditions and, more generally, for integration of high resolution biological AFM with many powerful optical techniques that require non-birefringent substrates.

Atomic force microscopy (AFM) has emerged as an important tool for macromolecular characterization in biological settings and is well suited for studying membrane proteins, which are challenging to address using traditional techniques^1^,^2^,^3^. Employing a vanishingly sharp force probe affixed to a precise translation stage, an AFM is capable of imaging membrane proteins without resorting to freezing or crystallization. Operating in physiological salt solution without the addition of any labeling, AFM resolves protein protrusions above the lipid bilayer, revealing macromolecular structure and conformational dynamics in near-native conditions. Despite unique capabilities, AFM has yet to reach its full potential within the nanoscience research community due to its lack of seamless integration with advanced light microscopy methods^4^.

Optical microscopy and spectroscopy tools are among the most broadly applied methods in biology. Common applications range from high throughput drug discovery assays based on fluorescence polarization^5^ to fundamental biophysical studies utilizing super-resolution methods that routinely break the diffraction limit^6^,^7^. Increasingly, optical microscopy techniques are being incorporated into AFM instruments to enhance functionality as well as precision^8^,^9^,^10^,^11^,^12^,^13^,^14^,^15^,^16^. Local probe techniques are not able to resolve small molecules in solution. A combined AFM-single molecule florescence microscope^14^ holds the potential to correlate ligand arrival with structural changes of a macromolecular target. Furthermore, AFM tips drift in space over time and experience forces in three dimensions. Inspired by techniques from the optical trapping microscopy community^17^,^18^,^19^ we have recently demonstrated an ultra-stable AFM^10^ that minimizes positional drift as well as a means to directly observe three-dimensional tip-sample interactions^15^.

High resolution 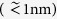 biological AFM imaging^2^,^20^,^21^ has been carried out nearly exclusively using freshly cleaved mica as a specimen supporting surface, with a handful of exceptions^22^,^23^,^24^,^25^. This is due to freshly cleaved mica’s inherent flatness, cleanliness, and biological compatibility. However, mica suffers from a fundamental limitation that has hindered its integration with numerous optical techniques. Mica exhibits biaxial birefringence; indeed, this naturally occurring material is used for optical wave plates. In general, propagation through birefringent material alters the polarization state and bifurcates the propagation direction of light in a manner which varies with material thickness. This makes it challenging to utilize freshly cleaved mica surfaces in modern optical systems, many of which employ highly focused and polarized laser beams passing through the specimen plane. Glass, on the other hand, is optically isotropic. It is a ubiquitous specimen supporting material for advanced optical microscopy methods^26^.

In this work we sought to couple the benefits of glass substrates with high resolution biological AFM. To obtain an AFM image, membrane proteins are held to the supporting surface through a lipid bilayer, thus allowing studies in near-native environments. Ideally, the underlying surface should be chemically inert and timely to prepare. Thus we explored alternative approaches to silanization which have been reported in pioneering work^22^,^27^,^28^. Using KOH-treated borosilicate glass cover slips as specimen supports, we demonstrate resolution of two integral membrane proteins at the level of monomer: bacteriorhodopsin, a bench mark sample in the field^29^, as well as SecYEG, the bacterial translocon from *E. coli*. Additionally, we observe the association of the ATPase SecA with SecYEG, forming a translocase at the membrane interface. We suggest more generally that glass-supported lipid bilayers may be an effective mimic of the situation *in vivo* wherein numerous punctate contacts are made with membrane, for example, by cytoskeletal elements^30^.

## Results and Discussion

**Figure 1:**
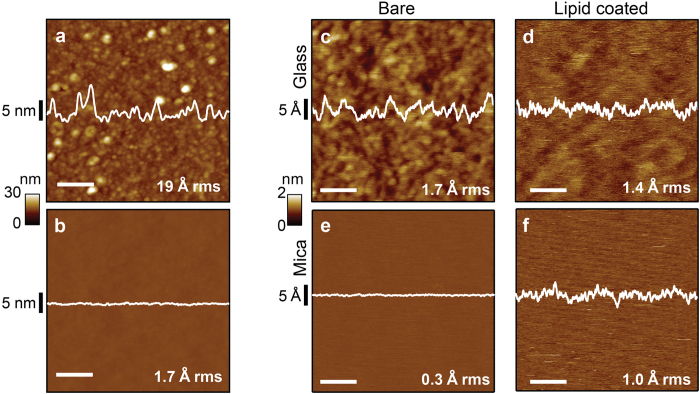
Glass preparation and reduction of roughness. Comparison of untreated glass (**a**) with KOH treated glass (**b**) reveals over an order of magnitude reduction in rms roughness. A further roughness reduction was observed when KOH treated glass (**c**) was coated with lipid (**d**). In contrast, images of mica before (**e**) and after (**f**) lipid deposition show increasing surface roughness upon lipid coating. Average rms roughnesses are indicated in the bottom right of each panel. Panels a & b share the same 30 nm vertical color scale. Vertical scales for data (**c**–**f**) are identical (2 nm) and indicated. Line scan profiles (*white traces*) are shown through the center of the images. Scale bars for (**a** & **b**) are 200 nm; for panels (**c**–**f**) bars are 20 nm.

As supplied by the manufacturer, borosilicate glass cover slips are rough on the molecular scale (Fig. 1a), exhibiting an average rms roughness of 19 ± 9.6 Å (mean ± S.D., evaluated over *N* = 100 non-overlapping 100 × 100 nm^2^ areas). This limits their direct application in high resolution AFM. Treatment in saturated KOH ethanol solution reduces the roughness by approximately an order of magnitude (Fig. 1b, roughness = 1.7 ± 0.3 Å, *N* = 440). We chose this approach because the etch rate of SiO_2_ is known to plateau and then to decrease at high KOH concentrations^31^; acting as a moderator, alcohol simultaneously reduces the etch rate and increases the uniformity^32^ of the etched surface (see Supplementary Information Fig. 1 for alternative treatment methods). Though smoother, KOH-treated glass is still approximately 6-fold rougher than freshly cleaved mica (Fig. 1e, roughness = 0.30 ± 0.03 Å, *N* = 127). The extremely flat nature of mica has advantages when carrying out imaging directly upon the solid-state surface, but our ultimate goal is to image membrane protein protrusions emanating from the upper leaflet of supported lipid bilayers.

Hence we explored the use of KOH-treated glass as a supporting surface for lipid bilayer imaging and compared results to those achieved with mica. Surprisingly, the difference in surface roughness between the upper bilayer leaflet imaged on KOH-treated glass and on mica is small (<2-fold; Fig. 1, compare panels d & f). This is noteworthy considering that untreated glass is approximately 60-fold rougher than mica itself. The effect comes about from two sources. First, the roughness of glass-supported samples is reduced, as can be seen when the same region is analyzed before and after deposition of *E. coli* polar lipid (Supplementary Information Fig. 2). Sampling of 340 non-overlapping areas reveals the average rms roughness is diminished from 1.7 ± 0.3 Å to 1.4 ± 0.4 Å (Fig. 1c,d, respectively). We attribute the observed smoothing to the bilayer’s ability to span local valleys in the complex topography of the glass surface. Second, in contrast to glass, the roughness of mica-supported samples increases upon lipid bilayer deposition to 1.0 ± 0.2 Å, *N* = 365 (Fig. 1, compare panels e & f). We attribute this roughening to lipid conformational fluctuations, which occur both laterally and vertically^33^, which also occur on glass, but which can only add disorder to the atomically-flat crystal plane of mica. Thus, for studying membrane protein protrusions, KOH-treated glass appears to be a suitable candidate for use as a supporting surface.

**Figure 2:**
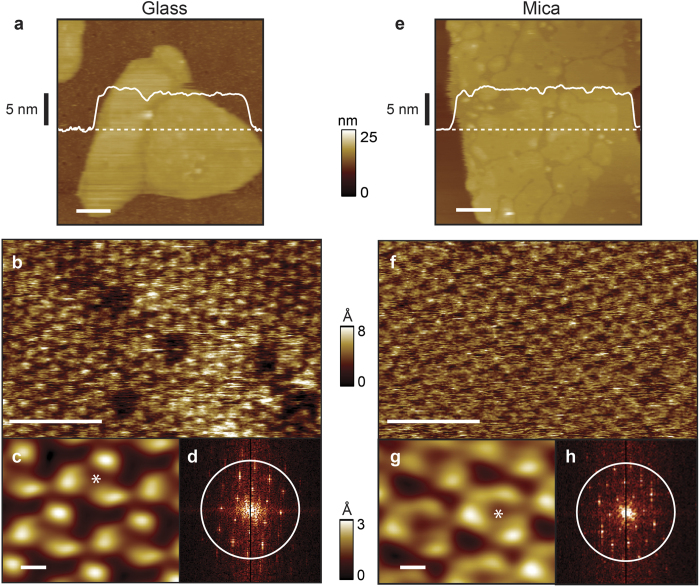
Molecular resolution imaging of bacteriorhodopsin on glass and comparison with mica. Large-scale image of purple membrane patch supported (**a**) by KOH-treated glass and (**e**) by mica. Smaller-scale imaging (**b**, on glass; **f**, on mica) reveals individual bacteriorhodopsin trimers. Correlation averaged and Fourier transformed data are shown (**c** & **d**, respectively, on glass; **g** & **h**, on mica). To facilitate direct comparison with glass substrates, data (**e**-**h**) was acquired using the same identical tip, but with a mica substrate. The asterisk in (**c** & **g**) indicates the center of the trimers. Scale bars are 200, 20 and 2 nm in (**a** & **e)**, (**b** & **f**), and (**c** & **g**), respectively. The vertical color scales for (**a** & **e**) and (**b** & **f**) are 25 nm and 8 Å, receptively. The vertical color scale for (**c** & **g**) is 3 Å.

To substantiate this notion we imaged bacteriorhodopsin from *Halobacterium salinarum* deposited on KOH-treated glass cover slips and compared the data to that acquired on mica (Fig. 2). Bacteriorhodopsin forms a well characterized two-dimensional lattice which has become an effective resolution standard for the field^29^. First, large scale AFM imaging was carried out to locate individual membrane patches, identified by their characteristic height (~5 nm) above the supporting glass surface (Fig. 2a). Smaller-scale imaging (Fig. 2b) revealed molecular resolution and periodicity inherent in the lattice. Correlation averaged data (Fig. 2c, *N* = 100 iterations) was used to determine the ~3.5 nm inter-trimeric distance, which is characteristic of the cytoplasmic side of bacteriorhodopsin^34^. Resolution achieved depends on a number of factors and can vary with individual tips within the same lot (SNL-A, Veeco)^29^,^34^. Therefore, the same identical tip that had been used with glass was used to image the same side of bacteriorhodopsin supported by mica (Fig. 2e–h). Two dimensional Fourier transforms of both data sets exhibit peaks out to and slightly beyond a 1 nm^−1^ radius (Fig. 2d,h) indicating that similar resolution was achieved on glass as on mica. Therefore, using this benchmark membrane protein sample, we demonstrated that there is no major difference in image fidelity over the areas required to visualize individual bacteriorhodopsin monomers.

There is a small difference in trimer conformation between the two samples, which were imaged in different buffer conditions (*glass*: 20 mM Tris, pH ~ 8.5, 200 mM KCl, 20 mM MgCl_2_; *mica*: 10 mM Tris, pH ~ 7.6, 150 mM KCl). The structure of bacteriorhodopsin depends strongly on the tip-sample interaction force as well as on the pH of the imaging buffer solution^35^,^36^. When the pH of the imaging buffers was made equal (pH ~ 8.5) the trimer conformations became more alike, although not identical (Supplementary Information Fig. 3a and b). It is possible that differing interactions between the two solid supporting surfaces and the proteins account for residual differences in the observed bacteriorhodopsin conformations. However, standard deviation maps generated from the correlation averaging revealed a similar magnitude of conformational dynamics (Supplementary Information Fig. 3c and d). This suggests that the underlying surface-protein interactions are not the primary cause of the conformational differences.

**Figure 3:**
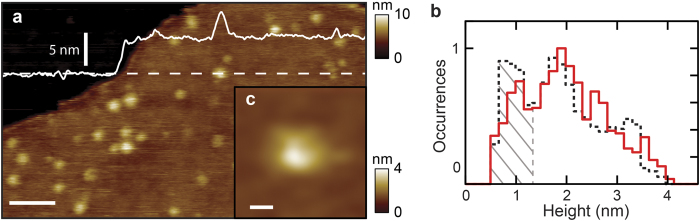
Visualization of SecYEG translocons in membrane. (**a**) AFM image of a glass-supported lipid bilayer containing SecYEG. A cross section profile (*white trace*) is also shown. Panel (**b**) shows height histograms of SecYEG on glass (*red, N* *=* 1203) and on mica (*dashed black, N* *=* 2766). Data was normalized to the total features, the fraction of occurrences in each bin of width 1.7 Å was plotted, the narrowest distribution was taken as the reference and the most highly populated bin was set to 1. An individual SecYEG monomer imaged on glass is shown (**c**). Scale bars are 100 nm, and 5 nm for panels (**a**) & (**c**), respectively.

To explore the potential of glass beyond two-dimensional arrays of membrane proteins, we studied individual components of the general secretory system of *E. coli*. We have previously characterized this system on mica surfaces, relating structural observations in near-native conditions to biological function^37^,^38^. Purified SecYEG translocons were reconstituted into liposomes and tested for translocation of precursor protein using established protocols^37^,^38^. Active proteoliposomes were then deposited onto KOH-treated glass surfaces for imaging. Individual translocons, identified as punctate protrusions (Fig. 3a,c), were classified by their heights above the lipid bilayer. Following previous work^38^, cytoplasmic and periplasmic protrusions were identified by exploiting the asymmetry inherent in the SecYEG structure^39^. The clear minimum in the height histogram at ~1.3 nm (Fig. 3b) separates the two orientations. The periplasmic orientation is indicated (Fig. 3b, *grey hatched*); cytoplasmic protrusions exhibit heights >1.3 nm. In agreement with our previous study using mica substrates (Fig. 3b, *black dashed*, data from ref. 38), there is a large distribution of heights for cytoplasmic SecYEG protrusions ranging from 1.3 to over 3 nm. This conformational diversity is likely to be due to dynamics of unstructured loops. There are two large (>30 amino acid) flexible loops connecting the ends of helices 6–7 and 8–9 of SecY^38^. Overall, these data indicate that the measured SecYEG protrusion topography is similar when imaged on glass and on mica.

**Figure 4:**
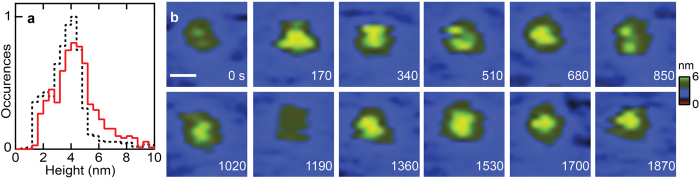
Direct observation SecA association with SecYEG. (**a**) Histograms of the maximum height of individual SecA/SecYEG complexes on mica (black dashed; N = 1088; bin size 4 Å) and on glass (red; N = 502; bin size 4 Å) surfaces. The fraction of occurrences in each bin was plotted, the narrowest distribution was taken as the reference and the most highly populated bin was set to 1. The prominent peak at ~4 nm is attributed to the height of the active SecYEG/SecA translocase and agrees well for data acquired on both surfaces. The peak between 1.5 and 3.0 nm corresponds to the height of the cytoplasmic protrusion of SecYEG in the absence of SecA. Periplasmic SecYEG protrusions which are 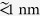 and do not bind SecA were excluded from analysis. (**b**) Tracking membrane activities for over 30 minutes reveals SecA association, disassociation, and re-association on glass-supported lipid bilayers. At t = 0 s the cytoplasmic SecYEG protrusion is visualized in the membrane. 170s later SecA binds, as indicated by the significant change in protrusion geometry. SecA dissociates at 1190 s. At 1360 s, SecA has re-associated with SecYEG. The scale bar is 10 nm.

The peripheral membrane protein SecA is known to cycle on and off the translocon at the membrane^37^, forming a SecYEG/SecA complex. To demonstrate that activities at membrane interfaces can be imaged using glass substrates, we prepared proteoliposomes by coassembly of SecYEG and SecA which results in a highly active form of SecYEG^37^, and tracked individual translocons for >1800s. The presence (or absence) of SecA engaged on the translocon can be determined by protrusion geometry (Fig. 4a)^37^. During the observation period, a molecule of SecA bound the cytoplasmic face of SecYEG at 170s (Fig. 4b), disassociated at 1190s, and then re-associated at 1360s, indicating that the translocon remains competent for SecA binding over more than 30 minutes of continuous imaging. Therefore, a local probe can track and directly visualize intricate protein-protein interactions occurring on glass-supported lipid bilayers for extended time periods.

Glass cover slips are among the most widely used specimen supporting surfaces and are an appealing non-birefringent specimen supporting surface for use in biological AFM. Their adoption would expand the promise of force microscope applications throughout nanoscale bioscience and biotechnology. However, glass is significantly rougher than mica. We show that after a straight-forward cleaning process followed by lipid deposition, the difference in roughness between the upper bilayer leaflet supported by glass and by mica is minor (<2-fold). Further, using two different integral membrane proteins, bacteriorhodopsin from *Halobacterium salinarum* and the translocon SecYEG from *Escherichia coli*, we demonstrate that glass cover slips can be used as effective substrates for AFM of membrane protein protrusions without introducing undue distortions or compromising resolution. Finally, direct visualization of SecA associating with the translocon during >1800s of observation demonstrates that glass-supported SecYEG remains in an active configuration as evidenced by its competency for binding this critical peripheral subunit.

Single molecule measurement techniques have produced powerful biophysical insights. A promising future direction in this field lies in the ability to bring complementary techniques to bear on a single biologically active macromolecular complex. Our work provides a path for incorporating advanced optical techniques into local probe studies in a timely manner, enabling, for example, precision measurements of membrane activities in near-native conditions.

## Methods

### Glass surface preparation

Glass coverslips purchased from Corning (18 × 18 mm, No. 1.5, catalog #: 2850-18) were used for the study. They were cleaned using KOH pellets (Sigma Aldrich, catalog #: P5958) dissolved in absolute ethanol (Fisher Scientific, catalog #: BP2818) as follows. Saturated KOH solution was prepared by mixing 90 g of KOH in 350 ml of absolute ethanol. This mixture was stirred using a magnetic stirrer in a 1 L beaker until the solution turned dark orange in color (~4 hrs). Home built Teflon baskets were used to hold the glass cover slips along their periphery for treatment in the saturated KOH solution for 3 min while immersed in a sonicator (Branson 5510). Coverslips were then rinsed with deionized water (18.2 MΩ*cm) using a squirt bottle and transferred into a beaker to be sonicated in distilled deionized water for an additional 3 min twice, with rinsing in-between. Coverslips were then rinsed with 95% ethanol, dried using ultra high purity nitrogen gas, and stored in a desiccator. Over several days surfaces can lose their hydrophilicity^40^. Thus, immediately before use, surfaces were plasma cleaned to render them hydrophilic as described below.

### AFM support design

Custom cut square coverslips (~13 × 13 mm) were attached to 12 mm diameter AFM specimen discs (TED PELLA, Product No. 16208) using epoxy (Devcon, part #: 20845). Care was taken to uniformly distribute the epoxy between the glass and specimen disc and to ensure it was devoid of air bubbles. Discs were left overnight for the glue to harden. Immediately prior to use, the support assemblies were plasma cleaned (Harrick Plasma PDC-001) in oxygen for 10 min at 250 mTorr using ~30 W forward RF power. The dimensions of glass coverslip were chosen to be slightly larger than that of the specimen disc, which minimizes exposure of the epoxy to the plasma as well as the imaging buffer solution.

### SecYEG and SecA purification

The translocon, SecYEG, was purified from a strain C43(DE3) suitable for over expression of membrane protein^41^ harboring a plasmid encoding *secY C329S*, *C385S*, *secE* with an N terminal His-tag, and *secG*^42^. Cells were broken by passage through a French pressure cell (8,000psi), and the membranes were isolated by centrifugation and solubilized in dodecyl-β-maltoside (DBM). SecYEG was purified by chromatography, using a HisTrap column (GE Healthcare), and stored at −80 °C in 20 mM Tris-Cl at pH 8, 0.3 M NaCl, 10% (wt/vol) glycerol, 0.6 mM DBM, and 2 mM DTT. SecA was purified as described^43^, with the following modifications: intact washed cells were incubated on ice for 30 min with 8 mM EDTA to chelate Mg2+ in the cell envelope. The cells were pelleted and washed twice to remove the EDTA before being lysed by three cycles of freezing and thawing in the presence of lysozyme. The removal of EDTA before lysis is crucial to prevent the extraction of zinc from SecA. After centrifugation, SecA was purified from the supernatant by chromatography, using a QAE (TosoHaas) column. The purified protein was dialyzed into 10 mM Hepes at pH 7.6, 0.3 M potassium acetate (KAc), 2 mM DTT, and stored at −80 °C. Concentrations of the proteins were determined spectrophotometrically at 280 nm, using coefficients of extinction as follows: SecA 78,900 M^−1^·cm^−1^; and SecYEG, 45,590 M^−1^·cm^−1^.

### Proteoliposome preparation

Proteoliposomes were prepared as described elsewhere^37^,^38^. Lipids (*E. coli* polar lipid extract, Avanti) in chloroform were blown dry with N_2_ and placed in a vacuum chamber overnight. A dry mechanical vacuum pump (XDS5, Edwards) was used to prevent backstreaming of oil, a potential contaminant. Dried lipids were suspended in 10 mM Hepes, pH 7.6, 30 mM KAc, 1 mM Mg(Ac)_2_. Unilamellar liposomes were prepared by extrusion through membranes (~100 nm pore diameter, Liposofast, Avestin). To form proteoliposomes the liposomes were swelled, but not disrupted, using a ratio of detergent to lipids of 4.65 mM DBM to 5 mM lipids^44^. After swelling for 3 h at room temperature, the proteins to be incorporated were added: SecYEG at 5 μM, and for coassembly of SecA, SecA at 5 μM dimer. Incubation was continued for 1 h at room temperature followed by addition of BioBeads SM-2 (BioRad) to remove the detergent. The proteoliposomes were isolated by centrifugation at 436,000 × g, 20 min. at 4 °C in a TL100.1 rotor (Beckman). The pellet was suspended in the same buffer and centrifuged again as above. The final pellet was suspended to give a concentration of approximately 8 mM lipid and 8 μM SecY. The suspension was stored at −80 °C.

### Bacteriorhodopsin preparation

Halobacterium salinarum strain S9 was grown and the purple membrane prepared as described^45^. The isolated purple membrane was suspended in distilled deionized water at 4.5 mg/ml bacteriorhodopsin. The concentration was determined using the extinction coefficient of the retinal chromophore at 568 nm (6.3 × 10^4^ M^−1^·cm^−1^) and molecular weight 26,000 for the protein. This stock solution was stored at −20 °C.

### AFM imaging

All AFM images were acquired in recording buffer at ~30 °C in tapping mode using a commercial instrument (Asylum Research, Cypher). Care was taken to control the magnitude of the tip sample force to 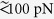 (estimated by comparing the free amplitude to the set point amplitude). Under such conditions, minimal protein distortion is expected^35^,^46^. Spring constants were determined using the thermal noise method. Details for each sample preparation follow. ***Glass alone***: The recording buffer was 10 mM HEPES pH 8.0, 200 mM KAc, 5 mM MgAc_2_; the tip used for the data shown in Fig. 1a,b was MSNL (Bruker) with spring constant ~0.4 N/m, a biolever mini (BL-AC40TS, Olympus) was used for Fig. 1c–f with spring constant ~0.06 N/m. ***Bacteriorhodopsin on glass***: A solution was prepared by diluting bacteriorhodopsin to 45 μg/ml in 10 mM Tris, pH ~ 7.8, 300 mM KCl buffer. Equal volumes of this solution and adsorption buffer (10 mM Tris, pH ~ 9.2, 700 mM KCl) were mixed before depositing onto a freshly cleaned glass support. After 1 hour incubation, the sample was rinsed with 10 volumes of recording buffer (20 mM Tris pH ~ 8.5, 200 mM KCl, 20 mM MgCl_2_). SNL (Veeco) tips with measured spring constant ~0.4 N/m were used. ***SecYEG and coassembled SecYEG/SecA complexes on glass***: Proteoliposome stock solutions were diluted to 80 nM SecYEG (or 80 nM coassembled SecYEG/SecA complexes), 80 μM lipid in recording buffer (10mM HEPES pH 8.0, 200 mM KAc, 5 mM MgAc_2_), immediately deposited on a freshly plasma cleaned glass support and incubated for ~20 minutes, followed by rinsing with recording buffer. Biolever mini tips (BL-AC40TS, Olympus) with measured spring constants ~0.06 N/m were used. ***Bacteriorhodopsin on mica***: Following established protocols^29^, equal volumes of stock solution and recording buffer (10 mM Tris pH ~ 7.6, 150 mM KCl) were mixed before depositing onto a freshly cleaved mica support. After a 1 hr incubation, the sample was rinsed with 10 volumes of recording buffer. SNL (Veeco) tips of measured spring constant ~0.4 N/m were used.

### Variability in glass surfaces

Some glass cover slips exhibit defects and a sparse distribution of pits is a common defect mode. The presence of small holes in the underlying supporting surface does not deleteriously effect the majority of topographic determinations of membrane protein protrusions^24^ (Supplementary Information Fig. 4).

### AFM image analysis

As is typical, images were flattened (≤2^nd^ order) to minimize background. To allow direct comparison of average root mean square (rms) roughness, all roughness calculations were carried out on 100 × 100 nm^2^ non-overlapping areas with the same pixel density (1.9 nm/pixel). Individual protein protrusions were cropped using custom software (Igor Pro, WaveMetrics) and a flood mask of ~2 Å above the lipid bilayer was applied to isolate protein protrusions. Software then extracted topographical data of individual protein protrusions above the bilayer. For the data shown in Fig. 3c and Fig. 4 we implemented tip deconvolution^47^,^48^. The program used blind tip estimation to determine the bluntest tip that could resolve the image. The generated tip geometry was then removed from the image, outputting a deconvolved image that more closely approximated the sample topography. Correlation averages (*N* = 100 iterations, Fig. 2c; *N* = 200 iterations, Fig. 2g) and standard deviation maps (Supplementary Information Fig. 3c and d) from the correlation averages were generated using SPIP software (Image Metrology).

## Additional Information

**How to cite this article**: Chada, N. *et al*. Glass is a Viable Substrate for Precision Force Microscopy of Membrane Proteins. *Sci. Rep*. **5**, 12550; doi: 10.1038/srep12550 (2015).

## Supplementary Material

Supplementary Information
