# Supplementary material for: Glass is a Viable Substrate for Precision Force Microscopy of Membrane Proteins
Source: Sci Rep. 2015 Jul 31;5:12550. doi: 10.1038/srep12550 (PMC4521160; doi:10.1038/srep12550)
Supplement: Supplementary Information [file srep12550-s1.pdf]

SUPPLEMENTARY INFORMATION

Glass is a viable substrate for precision force microscopy of membrane proteins

Nagaraju Chada[1], Krishna P. Sigdel[1], Raghavendar Reddy Sanganna Gari[1], Tina Rezaie Matin[1], Linda L. Randall[2] and Gavin M. King[1,2,]*

[1]Department of Physics and Astronomy, University of Missouri-Columbia, Columbia, Missouri 65211 USA

[2]Department of Biochemistry, University of Missouri-Columbia, Columbia, Missouri 65211 USA

*Corresponding author, email: kinggm@missouri.edu

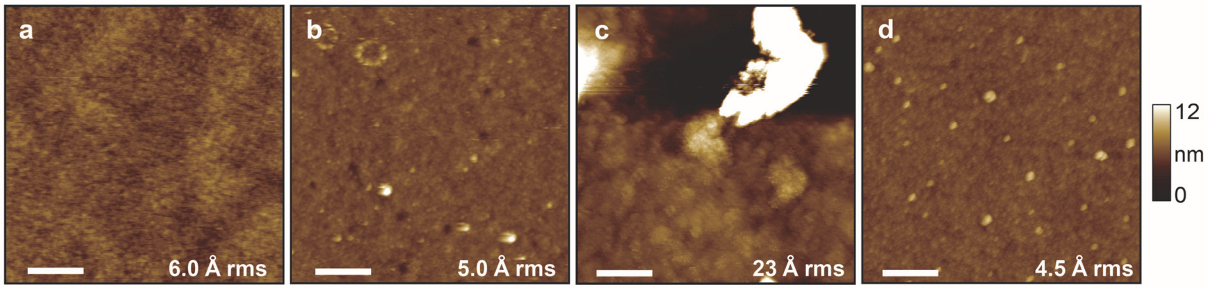

Supplementary Information Figure 1 | Comparison of several glass treatments.

Panel **a** shows an image of a glass coverslip after immersion in hydrofluoric acid (HF; 48 wt. %, Sigma Aldrich) for ~ 3 seconds. Panel **b** is an image of glass after treatment in buffered oxide etchant (BD solution; Transene, Inc.) for ~ 3 min. Panel **c** shows glass coverslip treated first in KOH solution as described in *Methods*, followed by HF solution for ~ 3 seconds. Panel **d** shows glass treated in saturated KOH solution as described in *Methods*, followed by dipping in buffered oxide etchant for ~ 3 min. All samples were rinsed in ethanol and distilled deionized water thoroughly prior to AFM investigation. Average rms roughnesses (evaluated in non-overlapping 100 X 100 nm^2 areas, $N \geq 100$) are indicated at the bottom right corner for each image. The standard deviations in roughness are 0.6, 2.2, 36.5, and 1.7 Å for panels a-d, respectively. Data acquired in recording buffer (10mM HEPES pH 8.0, 200 mM KAc, 5mM $MgAc_2$) using MSNL tips (Bruker). Lateral scale bars are 200 nm, the vertical color scale for all data is identical and indicated. For comparison, treatment methods described here yielded considerably rougher (> 2-fold) glass surfaces than saturated KOH solution alone, which produced an average rms roughness of 1.7 Å rms (see Fig. 1b & c in main manuscript).

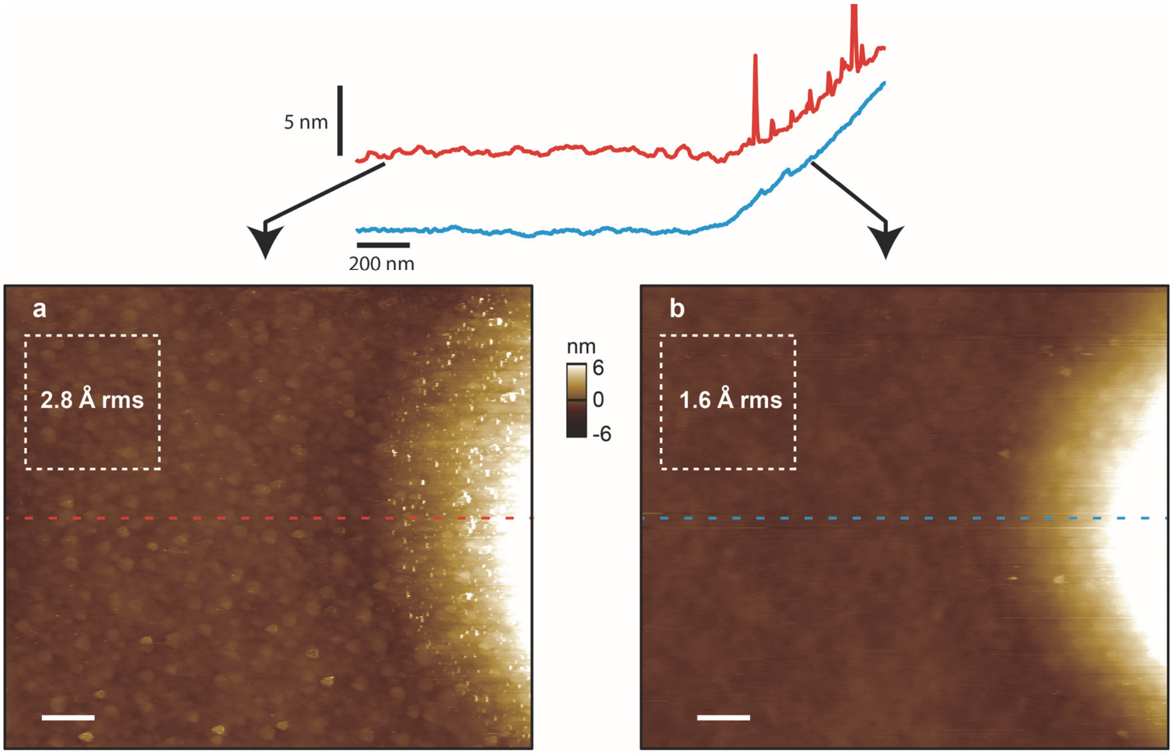

Supplementary Information Figure 2 | Direct visualization of the reduction of surface roughness via lipid deposition on glass.

AFM images of the same area of KOH treated glass before (**a**) and after (**b**) lipid deposition. Line scan profiles are shown above the images. The change in rms roughness determined in approximately the same area (*white dashed boxes*) is shown. A fiducial mark (visible on the right side of both images, deposited on the cover slip via physical vapor deposition of amorphous silicon through a shadow mask[1]) allows for image registration before and after lipid deposition. Data acquired in recording buffer (10mM HEPES pH 8.0, 200 mM KAc, 5mM $MgAc_2$) using biolever mini tips (BL-AC40TS, Olympus). Lateral scale bars are 200 nm, vertical color scales are identical and indicated.

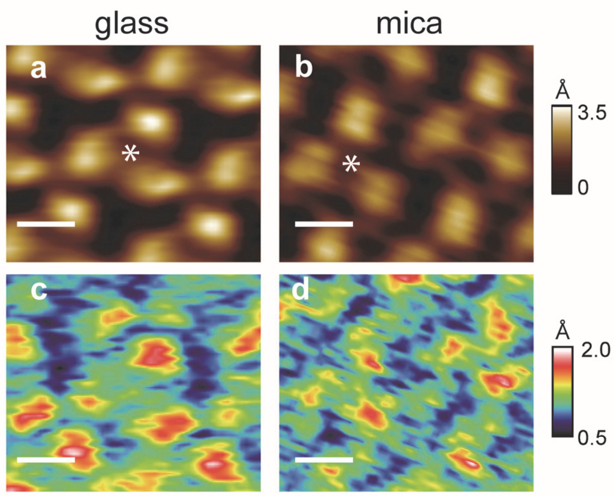

Supplementary Information Figure 3 | Bacteriorhodopsin conformation and conformational flexibility on glass and mica at pH 8.5.

Correlation averages of bacteriorhodopsin trimers on glass (a, N = 100 iterations) and on mica (b, N = 100). Bacteriorhodopsin assumes different conformations at different pH values[2-4] (compare Fig. 2g at pH 7.6 with Supplementary Information Fig. 3b at pH 8.5). Standard deviation maps of the correlation averages on glass (c) and mica (d) revealed a similar magnitude of conformational flexibility (~1.5 Å) suggesting that the underlying surface-protein interactions are similar. The asterisk in (a & b) indicates the center of the trimers. Imaging buffer conditions were as follows, *glass*: 20 mM Tris, pH ~ 8.5, 200 mM KCl, 20 mM $MgCl_2$; *mica*: 20 mM Tris, pH ~ 8.5, 150 mM KCl.

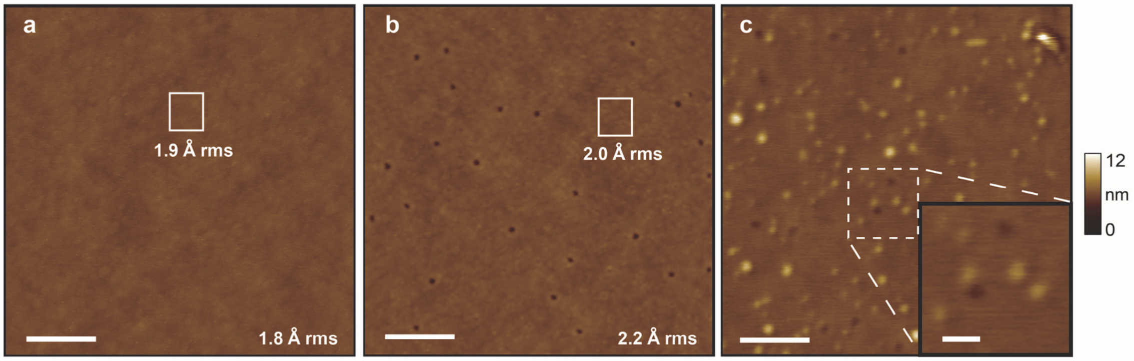

Supplementary Information Figure 4 | Glass substrates can accommodate membrane protein imaging in the presence of defects.

Glass cover slips can exhibit defects and a sparse distribution of pits is a common defect mode. Panels **a** and **b** show different glass coverlips treated with KOH solution as described in *Methods*. Though panel **b** exhibits a sparse distribution of pits it can still be used to extract useful data (as in ref. [5]). White boxes in panels **a** and **b** indicate 100 X 100 nm^2 areas with rms roughness of 1.9 and 2.0 Å, respectively. Average rms roughnesses are listed at the bottom right of panel **a** and **b** and were calculated over 100×100 nm^2 non-overlapping areas, $N \geq 100$; their standard deviations are 0.2 and 0.5 Å, respectively. Panel **c** shows a surface similar to panel **b** after deposition of liposomes containing SecYEG. The inset shows an expanded view indicating that the presence of holes in the underlying supporting surface does not interfere with topographic determinations of many protein protrusions. The scale bar for panels **a**, **b** and **c** is 200 nm and for the inset of panel **c** is 50 nm. Data was acquired in recording buffer (10mM HEPES pH 8.0, 200 mM KAc, 5mM $MgAc_2$). MSNL (Bruker) tips were used for panels **a** & **b**, a biolever mini tip (BL-AC40TS, Olympus) was used for **c**.

References:

1. Carter, A.R., King, G.M. & Perkins, T.T. Back-scattered detection provides atomic-scale localization precision, stability, and registration in 3D. *Optics Express* **15**, 13434-13445 (2007).
2. Müller, D.J., Buldt, G. & Engel, A. Force-induced conformational change of bacteriorhodopsin. *J Mol Biol* **249**, 239-243 (1995).
3. Müller, D.J. & Engel, A. Atomic force microscopy and spectroscopy of native membrane proteins. *Nat Protoc* **2**, 2191-2197 (2007).
4. Müller, D.J., Fotiadis, D. & Engel, A. Mapping flexible protein domains at subnanometer resolution with the atomic force microscope. *FEBS Lett* **430**, 105-111 (1998).
5. Goncalves, R.P., *et al.* Two-chamber AFM: probing membrane proteins separating two aqueous compartments. *Nat Methods* **3**, 1007-1012 (2006).
